# Public Access to Genome-Wide Data: Five Views on Balancing Research with Privacy and Protection

**DOI:** 10.1371/journal.pgen.1000665

**Published:** 2009-10-02

**Authors:** PG Consortium, George Church, Catherine Heeney, Naomi Hawkins, Jantina de Vries, Paula Boddington, Jane Kaye, Martin Bobrow, Bruce Weir

**Affiliations:** 1 Department of Genetics, Harvard Medical School, Cambridge, Massachusetts, United States of America; 2 The Ethox Centre, Department of Public Health and Primary Care, University of Oxford, Oxford, United Kingdom; 3 Department of Medical Genetics, University of Cambridge, Cambridge, United Kingdom; 4 Department of Biostatistics, University of Washington, Seattle, Washington, United States of America; The University of Queensland, Australia

## Introduction by Greg Gibson and Elizabeth Fisher

Just over twelve months ago, *PLoS Genetics* published a paper [1] demonstrating that, given genome-wide genotype data from an individual, it is, in principle, possible to ascertain whether that individual is a member of a larger group defined solely by aggregate genotype frequencies, such as a forensic sample or a cohort of participants in a genome-wide association study (GWAS). As a consequence, the National Institutes of Health (NIH) and Wellcome Trust agreed to shut down public access not just to individual genotype data but even to aggregate genotype frequency data from each study published using their funding. Reactions to this decision span the full breadth of opinion, from “too little, too late—the public trust has been breached” to “a heavy-handed bureaucratic response to a practically minimal risk that will unnecessarily inhibit scientific research.” Scientific concerns have also been raised over the conditions under which individual identity can truly be accurately determined from GWAS data. These concerns are addressed in two papers published in this month's issue of *PLoS Genetics* [2],[3]. We received several submissions on this topic and decided to assemble these viewpoints as a contribution to the debate and ask readers to contribute their thoughts through the PLoS online commentary features.

Five viewpoints are included. The Public Population Project in Genomics (P^3^G) is calling for a universal researcher ID with an access permit mechanism for bona fide researchers. The contribution by Catherine Heeney, Naomi Hawkins, Jantina de Vries, Paula Boddington, and Jane Kaye of the University of Oxford Ethox Centre outlines some of the concerns over possible misuse of individual identification in conjunction with medical and family history data, and points out that if geneticists mishandle public trust, it will backfire on their ability to conduct further research. George Church posits that actions directed toward restricting data access are likely to exclude researchers who might provide the most novel insights into the data and instead makes the argument that full disclosure and consent to the release of genomic information should be sought from study participants, rather than making difficult-to-guarantee promises of anonymity. Martin Bobrow weighs the risks and benefits and proposes four steps that represent a middle ground: Retain restricted access for now, make malicious de-identification practices illegal, increase public awareness of the issues, and encourage recognition that scientists have a special professional relationship of trust with study participants. Finally, Bruce Weir provides a commentary on the contribution of the two research articles from Braun et al. [2] and Visscher and Hill [3].

## P^3^G's Viewpoint: Future-Proofing Population Genomics?

The privacy concerns raised in the recent paper by Homer et al. [1] have had a significant impact on international open-access genomic databases. Individual single-nucleotide polymorphism (SNP) data from a participant in a GWAS can reveal whether that participant is in a DNA mixture including up to 1,000 participants. Although in hindsight it is clear that basic statistical theory would predict this to be the case, the reality is that it had previously gone completely unrecognised.

This situation illustrates the need to raise the level of discussion, thereby avoiding the ad hoc resolution of immediate privacy concerns and anticipating future scientific possibilities with a view to providing prospective guidance.

The implications of the Homer paper were discussed by the international Public Population Project in Genomics (P^3^G) (http://www.p3g.org). The consensus was that any scientist seeking to work with genomic data be required to adhere to an internationally agreed code of conduct and to provide proof of institutional status as a bona fide researcher.

A successful applicant could be awarded a permit and placed on a registry of users that would allow defined access to genomic databases (e.g., individually identifiable password and/or other criteria). This would avoid the need for repeated applications to prove bona fide status to different bodies, as is currently required. Infringement of the terms of the permit would bar the applicant from further access to genomic databases adhering to this code of conduct.

In the short term, interim models should be adopted that are broadly consistent with the principles of the proposed permit mechanism. Specifically: (1) determination of a researcher's bona fides may be internet based, but it *must* involve formal proof of institutional status; (2) each permit must be linked to an identified registered individual, using a universal “researcher ID” system such as the one already promoted by the GEN2PHEN project (http://www.gen2phen.org); and (3) large-scale secondary data providers should be expected to adhere to the same standards and practices as primary data providers when releasing data to a researcher holding a permit.

For this to work, it would require international recognition of the necessity and feasibility of such a strategy, as happened with the Bermuda Principles [4]. Such a proactive framework would contribute to sustaining ongoing public trust and participation in beneficial genomic research.

## George Church's Viewpoint: Considering a Creative Commons Universal Waiver

There is a clear movement to give all taxpayers access to government-funded research data [5]. We could debate amazingly sophisticated ways of encrypting public gene+trait data and even more amazing methods to thwart such encryption. However, we should acknowledge that the biggest security gaps are often social in nature. Getting researchers and their administrators to sign a legal form doesn't even begin to guarantee compliance. For example, high-security defense data access requires psychosocial security checks of relatives, past colleagues, and personality tests (far beyond NIH requirements). Nevertheless, authorized individuals occasionally take classified data outside of secure environments. Human gene+trait data seem destined to be amazingly useful, to be in huge demand, and to be capable of study from a vast number of angles. It is hard to guess who will make the biggest out-of-the-box analytical breakthroughs, but it is likely that these insights will come from highly integrative approaches, in which individuals are evaluated in cohorts holistically, just as a physician would—not as one disembodied organ plus one key SNP. It is likely that many of these insights will come from people outside of the clinical specialty of the original study (e.g., computer scientists and systems biologists). The more people granted access to these datasets, the more likely it is that someone will decide that it is ethically imperative (or cost-effective, or expedient) to share the data with researchers outside of the secure vault (or with the study participants/patients). The alternative model ([6], http://www.personalgenomes.org/) is to consent in advance with the understanding of full disclosure (not legalese weasel-words about “trying hard” to maintain anonymity). Furthermore, to help ensure informed consent rather than merely obtaining legal signatures on long consent forms, one can require 100% scores on tests of comprehension of the contents of the consent form and related materials. This has the side benefit of educating participants before the start of the study rather than after some potentially alarming result needs to be communicated back to them. Finally, the standard IRB (institutional review board) practice respects the autonomy of the individual research participant, hence does not require the consent of other family members. In the increasingly (re)identifiable datasets, increasing levels of family buy-in will likely be desirable, for example, in constructing pedigrees containing trait data. Fortunately there are many altruists who participate in medical research. However, one well-publicized incident of data leakage with inadequately informed consent could cause a backlash comparable to what happened with gene therapy in 1999 or Vioxx (rofecoxib) in 2004. In contrast, if we don't overpromise on anonymity and if these participants and their families are deeply engaged (not merely treated as de-identified animals) and they see direct value from these studies, then they might tell their stories widely and it may become increasingly easy to recruit more participants. The ability to make personal cell lines (http://www.personalgenomes.org/) and gene+trait data available under the new Creative Commons universal waiver, CC0 (http://creativecommons.org/weblog/entry/13304), could greatly enable unprecedented levels of commercial and academic creativity and collaborations.

## Catherine Heeney, Naomi Hawkins, Jantina de Vries, Paula Boddington, and Jane Kaye's Viewpoint: The Changing Context of Data Sharing—Types of Identifiability in Genomic Data

In 2003, a consortium of scientists working largely in public institutions triumphed over Celera in the race to map the human genome [7]. A willingness to make their data freely available on the Web played a part in this achievement. Subsequently, this approach to data sharing has become a norm in genomic research [8] and often a requirement of funding [9]. However, genomic information is not restricted to the research community. There are now private companies (for example, 23andMe) collecting sequence data and related information [10]. The real problem for genomic research is not that the information is available within the scientific research community, but that genomic sequence information is accessible to people *outside* of this community, who are not subject to the same safeguards, oversight, and professional codes of conduct. This has significant implications for our ability to protect the privacy of research participants.

Risks to privacy can arise because of the very nature of genomic sequence data [1],[11], but also because information can be inferred from other available data [12]. The ability to combine datasets exacerbates the problem [13]. For example, Gitschier demonstrates that by the iterative comparison of surnames in genealogical registries and data on Y chromosomes from the HapMap project, held in the CEU dataset, genetic information about named individuals could be inferred with considerable accuracy [13]. Moreover, Nyholt and colleagues show the difficulty of concealing sensitive genomic data by using linkage disequilibrium and data on other polymorphisms to infer information, which had not been directly released, about James Watson's *ApoE* gene [14].

Genomic data, combined with other information sources freely available on the Web, enables inferences about individuals, family members, or population groups that can undermine privacy. Inference is a reasonable stand-in for direct information and can even be used to support decisions about individuals, as it regularly is in the fields of insurance and credit [15]. The Internet supports access to everything, from Facebook to individual birth records, while ever-evolving data processing technologies enable efficient data collection and comparison of available datasets [16]. Raising these issues may appear alarmist; however, as Greenbaum et al. recently suggested, promising anonymity may already be a thing of the past due to the potential to infer information and the nature, amount, and variety of data freely available [17].

Requirements for the ethical management of research data have sought to balance the privacy of data subjects with the benefits of research, utilising anonymisation and informed consent [18]. However, it is now unrealistic to promise participants in research projects absolute confidentiality in relation to genomic data [19]. The rise in the sheer number of available data sources, in both the commercial and public sectors [20], coupled with the ease with which an individual's DNA can be (and is) analysed, suggests that the context for data release has changed. Disregarding this takes for granted the support of participants and the wider public for genomic research in a way that could have damaging consequences for future scientific endeavours.

## Martin Bobrow's Viewpoint: Toward a Middle Ground

Genuinely new ethical questions are rare, but difficult forms of old questions are exercising genomics researchers. Although posed as questions arising from GWAS or large cohort studies, the issues actually derive mainly from funders‚ pressure to encourage wide sharing of basic research data and from the power of the Internet as a tool for data sharing. If researchers simply kept their data to themselves, there would be no problem.

Making data available to many intelligent minds maximises the likelihood that the benefits of research will rapidly be returned to society, but also maximizes opportunity for breaching the duty of privacy to research participants. One way people attempted to reconcile these objectives was to make only aggregated genotype data publicly accessible on the Internet, with restricted access to data giving individual participants' genotypes. The landmark paper of Homer et al. shows, as is in retrospect intuitively obvious, that this doesn't work. The presence of an individual's DNA can be detected even if it is a very minor component of a mixture, and it is therefore possible that, under very special circumstances, an outsider could deduce that a named individual was part of, for example, a group of patients with a specific disease—the anonymised data could be re-identified.

To react to this surprising turn of events by abandoning large-scale genomic studies, or reducing the pressure for data sharing, would, in my view, be a disproportionate response to the level of threat as we currently see it. A widely discussed option is to get very explicit consent from patients. I would take it for granted that researchers must be open and honest with volunteer participants, but long, detailed technical consent documents tend to obfuscate, rather than illuminate, and may be better at shifting the legal burden than actually informing research participants. As such, they do little to engender the essential “trusting relationship” that should exist between the research community, research participants, and the public.

How, then, should we react to this new state of affairs?

1. Major research databases have already moved aggregated genomic data from open Internet access to restricted sites where researchers can gain access in return for undertakings on appropriate data use. Provided these mechanisms do not become overly onerous and that they can be given sufficient teeth to ensure that the obligations are enforced, this will not be a major obstacle to utilization of the data and could be retained indefinitely.
2. I wish it were clearer as to whether deliberately misusing data to re-establish the identity of anonymised individuals is a legally punishable offense. It breaches fundamental principles of data protection, but clear statements of penalties and the intention to enforce these would be extremely helpful. The law in any one country would not, of course, stop a malign individual in a distant jurisdiction, but it would seriously inhibit the use of his or her efforts by state agencies, police, insurers, and others in the data subject's own country, and without that there is little risk of harm.
3. The risk of harm to a research participant often comes not so much from their participation in the research, but from other activities, such as hospital data keeping, private sector genome studies, etc., which allow some genomic data associated with identifying characteristics of the individual to become accessible to the malign data miner. Without that, the “Homer” phenomenon has limited power to harm. Widespread public discussion to alert people and regulatory agencies to the dangers of having named genomic data lying in potentially accessible places is needed.
4. We all have confidential relationships with many people—lawyers, bankers, doctors—whom we trust because they are bound by professional codes of conduct with enforceable penalties. Research scientists, and particularly the institutions that employ them, probably need to make their own position in this regard more explicit—there should be clear codes of conduct and penalties for breaching them.

Further knee-jerk reactions should be avoided. More research will clarify exactly how sensitive these methods for re-identifying individuals are, particularly in relation to the choice of appropriate reference populations. I would hope the next 12 months would produce greater clarity and time to develop a proportionate long-term response.

## Bruce Weir's Viewpoint: Individual Genotyping in Forensics and GWAS Contexts

Braun et al. [2] and Visscher and Hill [3] have given helpful analyses of the question discussed by Homer et al. [1]: does an individual with a known genotype belong to a sample of individuals for which only allele frequencies are known? The analyses of all these authors suppose that allele frequencies are known also for a reference sample, and the question could be rephrased as: is this proband a member of this test sample of individuals, or of this reference sample, or neither sample? Homer et al. phrased their discussion in a forensic context where there is often interest in knowing whether or not a particular person was a contributor to a mixed sample of DNA from more than one person. They made brief mention of GWAS for which allele frequencies, but not individual genotypes, are made publicly available and where there may be interest in knowing whether or not a particular person was a member of a study. The resulting attention to this second situation, and the restriction of access to GWAS allele frequencies by the NIH and the Wellcome Trust, is likely the reason why Braun et al., as well as Visscher and Hill, concentrated on this situation and made only brief mention of forensic applications. Braun et al. employed both simulated and real data to show that the original analysis of Homer et al. is susceptible to linkage disequilibrium among the markers, the differences in allele frequencies between test and reference samples, and the relative sizes of these two samples. They also looked at the effects of the proband having a relative in either sample. Their work showed high specificity for the test statistic of Homer et al., but with the possibility of low sensitivity.

Although forensic science is not their main focus, Visscher and Hill introduce their treatment with likelihood ratios, as do forensic scientists in assessing how data support competing hypotheses. In this case, the two hypotheses are that the proband is either a member of the test sample or it is not in that sample. The ratio of the probabilities of the proband genotype under these two hypotheses is easy to formulate. If indeed the proband is in the test sample, Visscher and Hill show that the log-likelihood ratio has an expected value of 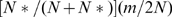, where *m* is the number of SNPs that are scored. The test sample has allele frequencies based on N individuals, and the reference sample has N* members. This value is multiplied by −1 if the proband is not a member of the test sample, and a test statistic can be constructed as the squared difference of the logarithms of the two probabilities divided by an estimate of the variance of the difference. In a very pretty result, Visscher and Hill show that this test statistic has an approximate expected value of 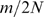. Good discrimination between the two hypotheses requires a large number of SNPs and/or a small test sample. Homer et al. also remarked on the advantage of using a large number of SNPs. The Visscher and Hill result assumes the SNPs are all independent, and they showed how linkage disequilibrium among the markers decreases the test statistic.

The relationship of a proband to a GWAS could be addressed by taking the cases as the test sample and the controls as the reference sample. Depending on whether the proband was a case, or a control, or neither, the log-likelihood ratios would be positive, negative or negative. Formal statistical tests follow from the work given by Visscher and Hill. Numerical work of all the authors confirms the wisdom of restricting access to GWAS case-control allele frequencies.

With the increasing sensitivity of forensic DNA profiling, often resulting from low template amplification [21], an increasing number of forensic samples contain DNA from multiple contributors, and the interpretation of such samples has progressed significantly since 1995, when lawyers could argue in US courts against the use of likelihood ratios for mixed samples [22]. Two features of multiple-contributor forensic profiles suggest the theory of Visscher and Hill will need further development for that context. In the first place, the very sensitivity of forensic genotyping means that allelic dropout is common [23], and fairly sophisticated methods [21] are needed to calculate likelihood ratios. Secondly, it will be some time before forensic scientists abandon the use of 13–20 microsatellite markers in favor of the very large numbers of SNPs considered by Braun et al. and by Visscher and Hill, largely because of the investment in very large offender databases. In June 2009, there were over seven million profiles in the US databases (http://www.fbi.gov/hq/lab/codis/clickmap.htm). Current forensic analyses start by determining whether or not all the proband alleles are seen within the mixture profile (maybe taking into account drop-out) and then calculating a likelihood ratio. Uncertainty over the number of contributors in the mixture (the test sample) will make allele frequency determination difficult for the test sample.

## Conclusion by Greg Gibson and Elizabeth Fisher

Something we can all agree on is that there is enormous goodwill in the general public toward medical research and a strong desire on the part of most people to be willing participants. At the same time, there is genuine fear of scientific abuse in general and gene technology in particular, and great potential for irreparable harm to both research and predictive health implementation, if identifiability issues are not addressed sensitively. As editors of a journal committed to open access to research, we are naturally suspicious of policies that restrict data access but we also understand that freedom usually comes at a price. What is essential is to get the balance of privacy protection and open, honest, and uniform consent right, and we hope that this short article encourages greater participation in the debate and education surrounding the issues.
